# Polysaccharides from the Coelomic Fluid of *Urechis unicinctus*: Extraction, Structural Diversity, and Potential Against Hypoxia

**DOI:** 10.3390/polym18101203

**Published:** 2026-05-14

**Authors:** Xiaodi Wang, Wenjie Wang, Rongfeng Li, Kun Gao, Ronge Xing, Xuexin Zhang, Gaoli Zhou, Lijing Yin, Junhao Chen, Hang Li, Guantian Li

**Affiliations:** 1 Laboratory of Experimental Marine Biology, Institute of Oceanology, Chinese Academy of Sciences, Qingdao 266000, China; 2 University of Chinese Academy of Sciences, Beijing 100049, China; 3 Laboratory for Marine Drugs and Bioproducts, Qingdao Marine Science and Technology Center, Qingdao 266237, China; 4 Laboratory for Marine Biology and Biotechnology, Qingdao Marine Science and Technology Center, Qingdao 266237, China

**Keywords:** sulfated polysaccharides, hypoxia, neuroprotection, marine natural products

## Abstract

The marine benthic invertebrate *Urechis unicinctus* exhibits extraordinary tolerance to hypoxic environments, making its coelomic fluid a unique and promising biological source for discovering novel stress-adapting macromolecules. Polysaccharides derived from the coelomic fluid of *U. unicinctus* were systematically extracted, fractionated, and characterized to investigate their structural features and associated biological activities. Gradient ethanol precipitation (30–80%) combined with DEAE-52 ion exchange chromatography yielded twelve fractions with distinct physicochemical properties. Significant variations were observed in molecular weight (10^3^–10^5^ Da), sulfate content (3.77–24.26%), and monosaccharide composition. High-ethanol fractions, particularly U68P and U18P (extracted at 60 °C and 100 °C, respectively, and both precipitated with 80% ethanol), were enriched in low-molecular-weight, highly sulfated heteropolysaccharides composed of galactose, fucose, glucosamine, and ribose. These fractions exhibited superior antioxidant activities, including strong scavenging effects against DPPH, ABTS, and hydroxyl radicals. Moreover, they demonstrated pronounced neuroprotective effects in the oxygen–glucose deprivation/reoxygenation (OGD/R) model using SH-SY5Y cells, significantly improving cell viability. Structure–activity relationship analysis revealed that reduced molecular weight, increased sulfation degree, and more diverse monosaccharide composition (e.g., more diverse monosaccharide composition) synergistically contribute to improved bioactivity by facilitating cellular uptake and exposing functional groups. In contrast, high-molecular-weight homoglucan fractions showed relatively weak effects. Overall, this study identifies *U. unicinctus* coelomic fluid as a promising source of bioactive polysaccharides and provides a theoretical basis for the development of marine-derived anti-hypoxic and antioxidant agents.

## 1. Introduction

Hypoxia, defined as a state of insufficient oxygen supply to tissues or cells, is a primary pathogenic factor in various critical conditions, including high-altitude sickness, cardiovascular diseases, and ischemic stroke [1]. Because the brain accounts for approximately 20% of total body oxygen consumption yet possesses limited antioxidant reserves, neurons are especially vulnerable to hypoxic insult [2]. Despite decades of investigation, clinically effective neuroprotective agents for hypoxia injury remain scarce, creating an urgent need for novel therapeutic candidates with well-characterized mechanisms of action [3].

In recent years, marine invertebrate-derived polysaccharides have garnered extensive attention as a structurally diverse reservoir of bioactive macromolecules. Unlike their terrestrial counterparts, marine-sourced polysaccharides often possess unique structural motifs, including sulfated galactans, fucoidans, and glycosaminoglycan-like chains, which contribute to their robust pharmacological profiles [4,5,6]. The bioactivities of these sulfated polysaccharides have been broadly documented across several marine taxa; for instance, polysaccharides from sea urchins have demonstrated potent antitumor properties [4], while those from *Sipunculus nudus* exhibit significant radioprotective [7,8,9], anti-fatigue [10], and immunomodulatory effects [11,12]. Similarly, sea cucumber polysaccharides are well-recognized for their anticoagulant [13], hypoglycemic [14], and hepatoprotective activities [15]. While the general properties of marine polysaccharides are established, their specific neuroprotective efficacy against hypoxia-induced damage has been less investigated.

*Urechis unicinctus* (also known as *haichang* or “sea intestine”), widely distributed in the intertidal zones and shallow muddy seabed of the Northwest Pacific coast (notably the Yellow Sea and Bohai Sea in China), has long been consumed as an edible marine resource [16]. This species inhabits U-shaped burrows in anoxic sediments, where it routinely experiences intermittent hypoxia and elevated hydrogen sulfide concentrations. It has been reported that the *U. unicinctus* could tolerate low oxygen levels (0.34 to 0.45 mg/L) for more than 46 h [17]. To adapt to such extreme conditions, *U. unicinctus* has evolved highly specialized physiological stress tolerance mechanisms. The coelomic fluid, serving as both the circulatory and respiratory medium in this coelomate organism, functions for gas exchange, nutrient transport, and redox homeostasis under hypoxic stress [18,19]. This distinctive biological role makes the coelomic fluid a potent, compelling matrix for the discovery of anti-hypoxic molecules.

Previous investigations of *U. unicinctus* have mainly focused on polysaccharides and glycosaminoglycans extracted from the body wall, revealing a range of bioactivities, including anticoagulant [20,21], hypoglycemic [22], anti-lipid peroxidation [23], and anti-platelet aggregation [24,25,26] activities. By contrast, the coelomic fluid, often discarded as a processing byproduct during commercial utilization, has received comparatively little attention. While recent studies have utilized *U. unicinctus* coelomic fluid for immunological research regarding antibacterial responses under environmental stress [27,28], there are currently no investigations addressing the extraction, structural characterization, or pharmacological evaluation of its polysaccharide components. Meanwhile, there are currently no reports on the anti-hypoxia properties of polysaccharides from *U. unicinctus*.

Therefore, the aims of this study were: (1) to establish a systematic fractionation strategy for isolating polysaccharides from the coelomic fluid of *U. unicinctus*; (2) to comprehensively characterize the chemical composition, molecular weight distribution, monosaccharide profiles, and spectroscopic features of the resulting polysaccharides; and (3) to evaluate the antioxidant and antihypoxia activities of these fractions using in vitro assays and human neuroblastoma cell models. The current research will establish a clear structure–activity relationship of *U. unicinctus* polysaccharides and provide a rational basis for the targeted development of marine-derived anti-hypoxic therapeutics.

## 2. Materials and Methods

### 2.1. Materials and Reagents

Live specimens of *U. unicinctus* (8–12 cm) were purchased from the Chengyang Seafood Wholesale Market (Qingdao, China) in October 2024. The animals were maintained in sterile seawater for 24 h to allow evacuation of intestinal contents. The human neuroblastoma cell line (SH-SY5Y) was procured from the Shanghai Cell Bank of the Chinese Academy of Sciences (Shanghai, China). 1,1-diphenyl-2-picrylhydrazyl (DPPH) and 2,2′-azino-bis(3-ethylbenzothiazoline-6-sulfonic acid) were provided by Aladdin Biochemical Technology (Shanghai, China). All other reagents were of analytical grade.

### 2.2. Extraction and Purification of Polysaccharides

After cleaning the body surface of approximately 2.5 kg of *U. unicinctus*, the body wall was punctured to collect a total of 4000 mL of the coelomic fluid. The coelomic fluid was diluted with ultrapure water at a ratio of 1:3 (*v*/*v*) and homogenized. The homogenate was divided into two groups and extracted in thermostatic water baths at 60 °C and 100 °C for 6 h, respectively. The extraction was repeated three times, and the supernatants were combined.

A sequential multi-enzyme hydrolysis protocol was employed to remove proteins while preserving polysaccharide integrity. Trypsin, papain, neutral protease, and alkaline protease (0.2%, *w*/*w*) were sequentially added to the supernatant. Before each addition, the system was adjusted to the optimal pH and temperature for the respective enzyme, each reaction step lasting for 6 h. Upon completion, the system was incubated in a boiling water bath (100 °C) for 15 min to inactivate the enzymes, followed by centrifugation at 13,000× *g* for 20 min to precipitate the denatured proteins. The resulting supernatant was concentrated using a rotary evaporator. Absolute ethanol was sequentially added to achieve final ethanol volume fractions of 30%, 50%, and 80% (*v*/*v*). At each gradient step, the system was allowed to stand overnight at 4 °C. The resulting precipitates were collected by centrifugation, redissolved in ultrapure water, desalted by dialysis for 72 h, and lyophilized. The fractions were designated as U63, U65, and U68 (from the 60 °C extraction group) and U13, U15, and U18 (from the 100 °C extraction group).

### 2.3. Anion Exchange Chromatography

The crude polysaccharide fractions were dissolved in ultrapure water (5 mg/mL), filtered through a 0.45 μm microporous membrane, and loaded onto an ÄKTA avant 25 system (Cytiva, Uppsala, Sweden) equipped with a Cellulose DEAE-52 column. The unretained neutral polysaccharides (designated N fractions) were eluted isocratically with ultrapure water at a flow rate of 2.0 mL/min. Subsequently, acidic polysaccharides (designated P fractions) were eluted with a linear gradient of 0–0.5 mol/L NaCl at the same flow rate. The elution profile was monitored offline by the phenol–sulfuric acid method (absorbance at 490 nm). The eluates corresponding to the main peaks were combined, desalted via dialysis, and lyophilized, yielding a total of 12 purified polysaccharide fractions (6 N + 6 P fractions).

### 2.4. Chemical Composition Analysis

The total carbohydrate content was determined by the phenol–sulfuric acid method, using D-glucose as the standard. Briefly, 2 mL of the polysaccharide solution was mixed with 1.5 mL of 5% phenol solution and 8 mL of concentrated sulfuric acid. After incubation for 30 min, the absorbance was measured at 490 nm using a Spark Cyto multimode microplate reader (Tecan Austria GmbH, Grödig, Austria). The protein content was measured using a BCA protein assay kit (Yuanye, Shanghai, China) according to the manufacturer’s instructions. Bovine serum albumin (BSA) served as the standard, and the absorbance was recorded at 562 nm. The sulfate content was evaluated via the barium chloride–gelatin turbidimetric method [29]. Polysaccharide samples were dissolved in 1 mol/L HCl and hydrolyzed at 100 °C for 6 h. The neutralized hydrolysate was reacted with a gelatin–BaCl_2_ complex reagent, and the absorbance was measured at 360 nm using K_2_SO_4_ as the standard to calculate the mass fraction of sulfate groups.

### 2.5. Molecular Weight Determination

The weight-average molecular weight (*M*_w_), number-average molecular weight (*M*_n_), peak molecular weight (*M*_p_), and polydispersity index (PDI = *M*_w_/*M*_n_) of each fraction were determined by an HPLC system (Agilent 1260 Infinity II, Agilent Technologies, Santa Clara, CA, USA) equipped with a TSKgel G3000SWXL column (7.5 mm × 300 mm) and a refractive index detector (RID). The mobile phase was a 50 mmol/L Na_2_SO_4_ solution with a flow rate of 0.6 mL/min at a column temperature of 40 °C. Dextrorotatory glycoside was used for calibration.

### 2.6. Monosaccharide Composition Analysis

Polysaccharide samples (5 mg) were hydrolyzed with 4 mol/L trifluoroacetic acid (TFA) at 115 °C for 4 h in sealed vessels. The neutralized hydrolysates were analyzed using an HPAEC-PAD ICS-5000+ system (Thermo Fisher Scientific, Sunnyvale, CA, USA) equipped with a Dionex™ CarboPac™ PA20 column with guard column. A gradient elution system consisting of ultrapure water, 200 mM NaOH, and a mixture of 200 mM NaOH and 500 mM sodium acetate was utilized as the mobile phase.

### 2.7. Spectroscopic and Morphological Characterization

#### 2.7.1. Ultraviolet–Visible (UV–Vis) Spectroscopy

Polysaccharide solutions (1 mg/mL in ultrapure water) were scanned over a wavelength range of 200–800 nm using a Spark Cyto multimode microplate reader (Tecan Austria GmbH, Grödig, Austria).

#### 2.7.2. Fourier Transform Infrared (FT-IR) Spectroscopy

FT-IR spectra were acquired on a Nicolet iS50 spectrometer (Thermo Fisher Scientific, Madison, WI, USA) over the range 4000–400 cm^−1^ at a resolution of 4 cm^−1^ with 32 co-added scans, equipped with an attenuated total reflectance (ATR) accessory.

#### 2.7.3. Nuclear Magnetic Resonance (NMR) Spectroscopy

Each lyophilized polysaccharide fraction (∼30 mg) was dissolved in 0.6 mL D_2_O (99.96 atom % D; Sigma-Aldrich, St. Louis, MO, USA) and subjected to three cycles of freeze–thaw–lyophilization to exchange labile protons. ^1^H and ^13^C NMR spectra were recorded on Varian VNMRS 500 MHz (Varian Inc., Palo Alto, CA, USA) and JEOL ECA-600 (JEOL, Akishima, Japan) spectrometers. Chemical shifts (δ) are reported in ppm relative to the residual D_2_O signal. Spectral data were processed and analyzed using MestReNova 14.0 software (Mestrelab Research S.L., Santiago de Compostela, Spain).

#### 2.7.4. Scanning Electron Microscopy (SEM)

The microscopic morphology of each lyophilized polysaccharide fraction was examined using a Zeiss GeminiSEM 500 field-emission scanning electron microscope (Carl Zeiss, Oberkochen, Germany). Samples were imaged at an accelerating voltage of 3.0 kV.

### 2.8. Antioxidant Assessment

The antioxidant activities of the 12 purified polysaccharide fractions were evaluated by in vitro radical scavenging assays. The half-maximal inhibitory concentration (IC_50_) was calculated by nonlinear regression.

#### 2.8.1. DPPH Radical Scavenging Activity

Gradient polysaccharide solutions were mixed with an equal volume of 0.1 mmol/L DPPH in ethanol. The mixture was reacted in the dark at room temperature for 30 min, and its absorbance was measured at 517 nm.

#### 2.8.2. ABTS Radical Scavenging Activity

Gradient polysaccharide solutions were mixed with a pre-activated ABTS^+^ working solution prepared from 7 mmol/L ABTS and 2.6 mmol/L K_2_S_2_O_8_. The reaction mixtures were incubated at room temperature for 10 min, and their absorbance was then measured at 734 nm.

#### 2.8.3. ·OH Scavenging Activity

Evaluated via the Fenton reaction. Gradient polysaccharide solutions were mixed with 1 mmol/L FeSO_4_, 6 mmol/L salicylic acid–ethanol, and 3% H_2_O_2_. The mixture was incubated at 37 °C for 30 min. The reaction was terminated by adding trichloroacetic acid (TCA), and the absorbance was measured at 510 nm.

### 2.9. Cytotoxicity and Anti-Hypoxic Assessment

#### 2.9.1. Cytotoxicity Assessment

Cell viability was quantitatively determined using the CCK-8 (Cell Counting Kit-8) assay. SH-SY5Y cells in the logarithmic growth phase were seeded into 96-well plates at a density of 1 × 10^4^ cells/well (100 μL/well) and incubated for 24 h to allow for attachment and metabolic stabilization. The original medium was then replaced with 100 μL of complete medium containing a series of concentrations of purified polysaccharides. Control groups (untreated cells) were established concurrently with multiple replicates per group. After an additional 24 h of incubation, the CCK-8 reagent was added to each well according to the manufacturer’s protocol. Absorbance was measured at 450 nm using a microplate reader.

#### 2.9.2. Anti-Hypoxic Effect Assessment

The anti-hypoxic effects of the polysaccharides were evaluated using an oxygen-glucose deprivation (OGD) model. SH-SY5Y cells were seeded into 96-well plates at 1 × 10^4^ cells/well and cultured for 12 h. The experimental groups were designated as follows: control, model, positive control group (edaravone, 80 μM), and polysaccharide-treated groups. Cells in the treatment and control groups were pre-treated with 100 μL of the respective media for 12 h. Following pre-treatment, cells were washed twice with glucose-free medium. Subsequently, 100 μL of glucose-free medium (containing the respective samples) was added to the model and treatment groups. These plates were placed in a hypoxia chamber (95% N_2_/5% CO_2_) for 8 h of strict hypoxic incubation. To simulate reoxygenation, the glucose-free medium was replaced with normal glucose-containing medium (with samples), and cells were incubated under normoxic conditions for another 6 h. Finally, cell survival rates were measured using the CCK-8 assay.

### 2.10. Data Analysis

All quantitative experiments were independently repeated at least three times (n ≥ 3), and the data are presented as the mean ± standard deviation (SD). Statistical analyses were performed using SPSS 27 software (IBM Corp., Armonk, NY, USA). Differences among groups were evaluated via one-way analysis of variance (ANOVA), followed by Duncan’s multiple range test for post hoc analysis. A value of *p* < 0.05 was considered statistically significant. Experimental graphs were plotted using OriginPro 2022 software (OriginLab Corporation, Northampton, MA, USA). NMR spectral data were processed and analyzed using MestReNova 14.0 software (Mestrelab Research S.L., Santiago de Compostela, Spain). The acquisition and integration of chromatographic data (HPLC and HPAEC-PAD) were executed via the Chromeleon Chromatography Data System (Thermo Fisher Scientific, Waltham, MA, USA).

## 3. Results

### 3.1. Fractional Extraction and Chromatographic Purification

The extraction parameters significantly influenced the solid–liquid phase distribution of the polysaccharides. Fractional precipitation using 30%, 50%, and 80% ethanol was performed following water extraction at 60 °C and 100 °C. As shown in Table 1, the overall absolute yields of all fractions were relatively low (1.51–3.80‰, based on wet coelomic fluid weight). At 80% ethanol precipitation, increasing the extraction temperature from 60 °C to 100 °C decreased the yield from 2.85‰ to 1.51‰, indicating potential thermal degradation of the polysaccharide backbone [30]. However, the 100 °C extraction exhibited superior deproteinization efficacy (residual protein: 0.87–1.23%) and high carbohydrate purity (90.94–95.68%). Although the apparent yield is low, primarily due to the extreme moisture content of the raw coelomic fluid (>90%), it is not anticipated to be a barrier to future industrial scale-up. Since coelomic fluid is a non-edible byproduct discarded during *U. unicinctus* processing, its utilization represents a zero-cost feedstock that aligns well with circular economy principles. Furthermore, future commercial-scale operations can readily employ standard high-capacity membrane filtration or spray-drying techniques to efficiently manage large liquid volumes, ensuring that the initial low solute concentration does not impede practical viability.

**Figure 1:**
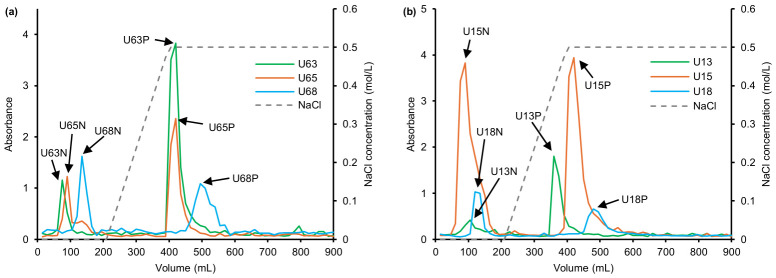
Ion chromatograms of the crude polysaccharides obtained with different extraction temperatures: (**a**) 60 °C and (**b**) 100 °C. Note: U63, U65, and U68 represent those extracted at 60 °C and obtained by alcohol precipitation at concentrations of 30%, 50%, and 80%, respectively; U13, U15, and U18 represent those extracted at 100 °C and obtained by alcohol precipitation at concentrations of 30%, 50%, and 80%, respectively. N and P denote the neutral and acidic (polar) polysaccharide fractions, respectively.

To elucidate the distribution of polar segments, the crude polysaccharides were fractionated using Cellulose DEAE-52 anion exchange chromatography (Figure 1). Water and NaCl elution yielded neutral/weakly polar (N fractions) and acidic (P fractions) polysaccharides, respectively. In the mild 60 °C system, the 30% ethanol-precipitated fraction (U63) exhibited a prominent acidic peak, whereas the 80% fraction (U68) transitioned towards low-molecular-weight components rich in highly hydrophilic neutral segments. Elevating the extraction temperature to 100 °C induced a distinct structural shift. The macromolecular backbone that predominantly precipitates at 30% ethanol was diminished, rendering the 50% fraction (U15) the dominant chromatographic phase. Furthermore, as the ethanol concentration increased from 30% to 80%, the decreasing dielectric constant of the solvent mixture [31] led to a significant increase in the proportion of N fractions (from 10.78% to 38.06% at 60 °C).

Notably, the high-ethanol fractions (e.g., U68 and U18) exhibited lower overall absorbances. This is likely due to the inherent low sensitivity of the classical phenol–sulfuric acid method towards high-density anionic groups or amino sugars. To prevent the omission of bioactive components due to methodological biases, all 12 purified fractions were retained for subsequent structural characterization.

### 3.2. Quantitative Analysis of Sulfate Content

**Figure 2:**
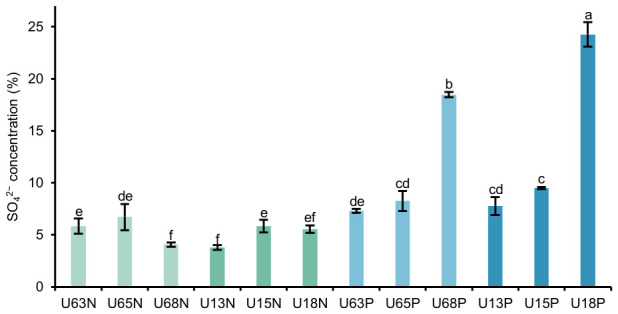
Sulfate content of polysaccharides from the coelomic fluid of *U. unicinctus*. Note: U63, U65, and U68 represent those extracted at 60 °C and obtained by alcohol precipitation at concentrations of 30%, 50%, and 80%, respectively; U13, U15, and U18 represent those extracted at 100 °C and obtained by alcohol precipitation at concentrations of 30%, 50%, and 80%, respectively. N and P denote the neutral and acidic (polar) polysaccharide fractions, respectively, isolated by DEAE-52 cellulose column chromatography. Different lowercase letters above the bars indicate significant differences between groups (*p* < 0.05), while sharing the same letter indicates no significant difference.

The degree of sulfate modification significantly influences the conformation and biological activities of marine polysaccharides. As shown in Figure 2, the mass fractions of sulfate groups across the 12 polysaccharide fractions exhibited substantial heterogeneity, ranging from 3.77% to 24.26%. The acidic polysaccharides (P fractions) eluted with NaCl possessed significantly higher sulfate contents than their corresponding water-eluted neutral counterparts (N fractions). For instance, the sulfate content of U18P reached 24.26 ± 1.19%, which is comparable to the sulfation level (21.4%) of purified polysaccharides from the sea cucumber *Holothuria fuscopunctata*. This confirms that sulfate groups are primarily responsible for the strong anionic characteristics of the P fractions [32].

Furthermore, the ethanol concentration demonstrated a targeted regulatory effect on sulfate enrichment. In both the 60 °C and 100 °C extraction systems, increasing the ethanol concentration from 30% to 80% sharply decreased the dielectric constant of the solvent. This shift induced the phase separation of highly hydrophilic fragments carrying dense polar sulfate groups, resulting in a substantial increase in the sulfate contents of U68P (18.47%) and U18P (24.26%). This distinct high-sulfation modification not only reflects structural polymorphism but also generates strong intramolecular electrostatic repulsion due to the dense negative charge centers. Such repulsion likely forces the polysaccharide backbone into a more extended spatial conformation, facilitating the spatial exposure of active sites.

### 3.3. Monosaccharide Composition

Monosaccharide composition is a fundamental determinant of the higher-order conformation and pharmacological activities of polysaccharides. Quantitative HPAEC-PAD analysis revealed the significant regulatory effects of the ethanol precipitation gradient and chromatographic environment on the polysaccharide backbone. The coelomic fluid polysaccharides of *U. unicinctus* exhibited substantial structural heterogeneity, transitioning from homoglucans to highly specialized heteropolysaccharides (Table 2). Standard curves are presented in Figure S1.

Under water elution (N fractions) and low-to-medium ethanol precipitation (30–50%), the polysaccharide backbones demonstrated extremely high homogeneity. The molar proportions of glucose (Glc) in fractions such as U13N and U15N exceeded 99.50%. The corresponding acidic fractions (e.g., U63P, U15P) also retained a glucose-dominated backbone (abundance of 89.98–96.56%). This indicates that mild ethanol precipitation is an effective approach for enriching high-purity homoglucans from the coelomic fluid.

As the ethanol concentration increased to 80%, the monomer composition of the N fractions shifted significantly. The glucose proportions in U68N and U18N decreased to 72.92–76.86%, accompanied by the marked appearance of ribose, glucosamine (GlcN), and mannose. This structural enrichment confirms that a low-dielectric-constant environment effectively disrupts the solution stability of highly hydrated heteropolysaccharides, thereby facilitating the retention of complex heteropolysaccharide fragments characterized by higher branching degrees and shorter main chains [33].

Crucially, in the fractions obtained via 80% ethanol precipitation combined with high-salt elution (U68P and U18P), the glucose abundance dropped precipitously to below 10%. Instead, galactose (~32%), ribose (~21–24%), fucose (~13–14%), and glucosamine (~10%) became highly dominant. In marine biochemical structure–activity systems, complex backbones rich in galactose, fucose, and amino sugars are the typical monosaccharide fingerprints of highly active sulfated glycosaminoglycans or fucoidans [11,22]. The enrichment of these specific residues provides abundant attachment sites for dense sulfation modifications [34]. Such complex main/side-chain structures, along with the high-density sulfation they carry, are considered the structural basis triggering specific affinities between macromolecules and cellular receptors, thereby exerting high-order physiological functions [35].

### 3.4. SEM Morphological Analysis

**Figure 3:**
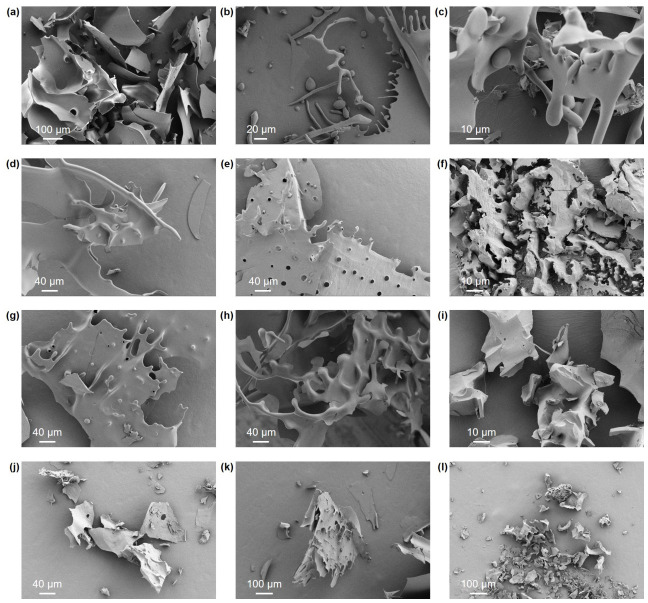
Scanning electron microscopy images of the lyophilized polysaccharide powders from the coelomic fluid of *U. unicinctus*. (**a**) U63N; (**b**) U65N; (**c**) U68N; (**d**) U13N; (**e**) U15N; (**f**) U18N; (**g**) U63P; (**h**) U65P; (**i**) U68P; (**j**) U13P; (**k**) U15P; (**l**) U18P. Note: U63, U65, and U68 represent those extracted at 60 °C and obtained by alcohol precipitation at concentrations of 30%, 50%, and 80%, respectively; U13, U15, and U18 represent those extracted at 100 °C and obtained by alcohol precipitation at concentrations of 30%, 50%, and 80%, respectively. N and P denote the neutral and acidic (polar) polysaccharide fractions, respectively.

As shown in Figure 3, the lyophilized solid states of the 12 fractions exhibited marked structural heterogeneity. The extraction temperature decisively influenced the macroscopic continuity of the polysaccharides. Fractions extracted under mild conditions at 60 °C (e.g., U63N and U63P) generally presented as large, continuous, and irregular lamellar structures. In contrast, those extracted at 100 °C (e.g., U13N and U13P) displayed distinct fragmentation, primarily appearing as sharp-edged, debris-like aggregates. This morphological difference further substantiates the earlier hypothesis of high-temperature-induced thermal depolymerization: the reduction in chain length significantly weakens the cohesion of the polysaccharide backbone, ultimately leading to a collapse in structural integrity.

Furthermore, the ethanol concentration gradient dictated the morphological transition from dense lamellae to loose granules. Taking the P fractions as an example, the 30% ethanol-precipitated fraction (U63P) exhibited a dense and continuous lamellar structure and the 50% fraction (U65P) developed curled dislocations on its surface, whereas the fractions precipitated in the extremely low-dielectric-constant 80% ethanol environment (e.g., U18N and U18P) evolved into very fine, discrete fragments. Conversely, the short-chain fragments precipitated at high ethanol concentrations lack physical chain entanglement. During the ice crystal sublimation phase of lyophilization, the polysaccharide aggregates cannot resist the intense capillary shrinkage stress generated by dehydration, thereby tending to form debris-like morphologies [36,37,38].

Finally, charge density and chemical modification states directly determined the development of the microscopic pore network in the solid state. Comparing the lyophilized powders of fractions obtained under identical extraction parameters, samples of acidic polysaccharides (e.g., U65P) universally exhibited a highly porous, honeycomb-like three-dimensional structure and sponge-like networks, whereas the neutral fractions (e.g., U65N) had relatively smooth and compact surface morphology. Considering the previously determined high sulfate content (up to 24.26%), this topological discrepancy originates from the strong intra- and intermolecular electrostatic repulsion induced by the high-density anionic modifications. During the freeze-drying process, this repulsive force effectively counteracts the volume shrinkage and agglomeration tendencies of the polymer chains, thereby supporting and maintaining a highly porous, stereoscopically rigid skeleton [39,40,41].

### 3.5. Molecular Weight and Distribution Characteristics

The relative molecular weight and its distribution are core parameters that determine the spatial conformation, hydrodynamic volume, and ultimate biological activities of polysaccharides. Using an HPLC platform, the molecular parameters of the 12 polysaccharide fractions from *U. unicinctus* coelomic fluid were systematically determined (Table 3). In highly polydisperse polymer systems, compared to the number-average molecular weight (*M*_n_), which is highly sensitive to trace small molecules, the weight-average molecular weight (*M*_w_) more accurately reflects the characteristics of the macromolecular backbone that constitutes the majority of the mass. Previous structure–activity relationship studies indicate that high-molecular-weight polysaccharides often face hindered transmembrane uptake due to their large excluded volumes; thus, molecular size profoundly and directly impacts their therapeutic efficacy [42]. Therefore, this study utilizes *M*_w_ and the polydispersity index (PDI), which reflects the molecular weight distribution width, as the primary evaluation metrics.

The molecular weight data revealed that the extraction temperature regulated the degradation of the macromolecular backbone. A horizontal comparison showed that the *M*_w_ of the high-temperature (100 °C) extraction group (U1 series) was significantly lower than that of the mild (60 °C) extraction group (U6 series). This pattern was consistent across the polar P fractions, further substantiating the earlier hypothesis that high temperatures induce irreversible cleavage and depolymerization of the polysaccharide main chain. Furthermore, the ethanol concentration demonstrated a definitive molecular weight cut-off effect on the phase separation kinetics of the fractions. The *M*_w_ of the 30% and 50% ethanol-precipitated fractions generally remained in the high-molecular-weight range of 1.12 × 10^5^ to 1.29 × 10^5^ Da, with PDIs ranging from 1.49 to 1.67, presenting the broad distribution typical of natural polysaccharides. However, when the ethanol concentration increased to 80%, the molecular parameters underwent a drastic shift, with both PDIs narrowing significantly to approximately 1.25.

This abrupt shift provides a strong mechanistic basis for understanding the fractionation behavior of polar heteropolysaccharides. In fractional precipitation, varying concentrations of ethanol physically precipitate polysaccharide fractions strictly in descending order of their molecular weights [43]. Lower-molecular-weight, highly sulfated oligosaccharides, owing to their minimal hydrodynamic volumes and dense surface charges, typically possess superior targeted tissue penetration and higher-order biological activities. The low-polydispersity fragments (e.g., U18P and U68P) captured by the 80% precipitation process not only eliminate the potential interference of macromolecular steric hindrance but also precisely isolate the core bioactive candidates for subsequent advanced pharmacological evaluations.

### 3.6. UV-Vis Spectroscopic Analysis

**Figure 4:**
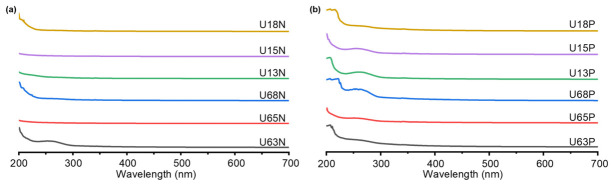
Full-spectrum scanning profiles of polysaccharides from the coelomic fluid of *U. unicinctus*. (**a**) Full-wavelength scanning spectra of neutral polysaccharide fractions extracted at 60 °C and 100 °C; (**b**) Full-wavelength scanning spectra of polar polysaccharide fractions extracted at 60 °C and 100 °C. Note: U63, U65, and U68 represent those extracted at 60 °C and obtained by alcohol precipitation at concentrations of 30%, 50%, and 80%, respectively; U13, U15, and U18 represent those extracted at 100 °C and obtained by alcohol precipitation at concentrations of 30%, 50%, and 80%, respectively. N and P denote the neutral and acidic (polar) polysaccharide fractions, respectively.

Full-wavelength UV–Vis scanning (200–700 nm) intuitively revealed the impacts of the extraction and purification processes on the optical purity and structural characteristics of the *U. unicinctus* coelomic fluid polysaccharides (Figure 4). No evident absorption peak was observed at 280 nm, indicating effective removal of proteins. The subtle peaks observed at 260 nm are attributed to trace nucleic acid residues, which become visible primarily due to the high sample concentration used during analysis (1 mg/mL). These weak signals became visually apparent primarily due to the relatively high sample concentration used during the analysis, which amplified the absorbance of minor impurities. Furthermore, all samples exhibited extremely low absorption and a stable baseline in the visible region (700 nm), excluding interferences from residual pigments and colloidal scattering. This confirms the excellent homogeneity of the purified polysaccharides in aqueous solutions [44].

In the far-ultraviolet region, the absorption features of the fractions diverged significantly, driven by the separation dielectric constant and charge density. The neutral N fractions eluted with pure water exhibited typical terminal absorption characteristics of macromolecular carbohydrates, with the maximum absorption localized near the detection limit of 200 nm. This originates primarily from the electronic transitions of free and hemiacetal hydroxyl groups on the polysaccharide backbone [45]. Specifically, the high-ethanol-precipitated fractions (U18N and U68N) displayed extremely narrow absorption bands that decayed exponentially toward longer wavelengths, indicating a low charge abundance and a lack of complex modifying groups. Conversely, the broadened baselines of the low-ethanol-precipitated fractions (U63N and U13N) suggest the potential supramolecular aggregation of these macromolecular backbones in aqueous solutions.

By contrast, the polar P fractions eluted with the NaCl gradient exhibited distinct redshift and broadening of the absorption bands. Notably, for U68P and U18P, which were specifically enriched at 80% ethanol precipitation, the maximum absorption peak redshifted significantly to the 220–222 nm range. In the spectroscopic systems of marine macromolecules, strong absorption in this band typically corresponds to electronic transitions (e.g., n → π* transitions) induced by heteroatoms rich in lone-pair electrons, such as the oxygen atoms in strongly polar sulfate groups or the nitrogen atoms in amino sugars [45,46]. This spectroscopic feature perfectly corroborates the aforementioned surge in sulfate mass fraction (up to 24.26%) and the high enrichment of glucosamine in the U68P/U18P fractions.

### 3.7. FT-IR Spectroscopic Analysis

**Figure 5:**
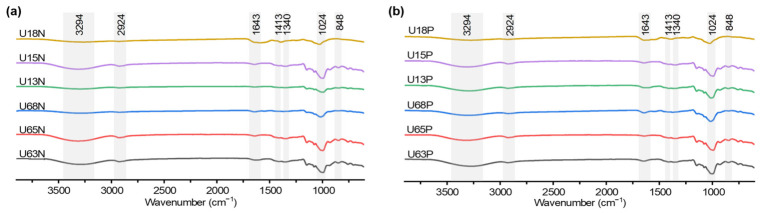
FT-IR spectrum analysis of polysaccharides from the coelomic fluid of *U. unicinctus*. (**a**) FT-IR spectra of neutral polysaccharide fractions extracted at 60 °C and 100 °C; (**b**) FT-IR spectra of polar polysaccharide fractions extracted at 60 °C and 100 °C.

The absorption profiles of all fractions exhibited highly similar characteristic bands across the entire frequency domain (Figure 5, Table S1). In the regions of 2924–2932 cm^−1^ and 1022–1029 cm^−1^, all samples displayed constant and prominent absorption, with no distinct differences in the fingerprint contours between the 100 °C and 60 °C extraction groups. The band near 2930 cm^−1^ is attributed to the C–H stretching vibration of the sugar ring [47]. The 1020 cm^−1^ band is primarily generated by the highly coupled stretching vibrations of the intra-ring C–O–C glycosidic bonds and side-chain C–O–H, which is a typical fingerprint region for macromolecular pyranose derivatives [42,48,49]. Additionally, a consistent absorption band was observed at approximately 1643 cm^−1^ across all spectra, which is assigned to the Amide I vibrations and/or the O–H bending modes of tightly adsorbed water [50]. These results indicate that although high-temperature extraction caused a significant decrease in the weight-average molecular weight, it did not disrupt the stable pyranose ring covalent structures.

Notably, a crucial spectral shift exists between the neutral N series and the polar P series. The broad O–H stretching vibration band of the neutral N polysaccharides was mainly distributed between 3294 and 3326 cm^−1^. Conversely, this absorption center in the polar P polysaccharides underwent a significant high-frequency blueshift, transitioning to the 3376–3390 cm^−1^ range. Previous conformational thermodynamic studies indicate that alterations in non-covalent electrostatic interactions within macromolecular systems significantly affect the vibration frequency of free hydroxyl groups [51]. Combined with the previously determined high sulfate abundance, the shift in the P fractions confirms the introduction of high-density anionic charges.

Furthermore, specific absorption bands in the fingerprint region (600–1300 cm^−1^) provided key insights for identifying the structural modifications of the polar polysaccharides. All polar P fractions exhibited strong characteristic absorption peaks in the sensitive intervals of 1236–1245 cm^−1^ and 842–852 cm^−1^. According to spectroscopic localization theory, the strong absorption near 1240 cm^−1^ is unambiguously assigned to the asymmetric stretching vibration of sulfate ester groups (S=O) [52,53]. Meanwhile, the characteristic absorption in the 850 cm^−1^ region not only indicates the anomeric carbon configuration of α-pyranose, but its vibrational frequency also highly coincides with the spatial signature of axial sulfate groups specifically substituting at the C-4 position of the pyranose ring [54]. Considering the earlier monosaccharide composition analysis, which showed that the P series is rich in galactose and fucose, the inference of C-4 sulfation modification exhibits high logical consistency.

### 3.8. NMR Spectroscopic Analysis

The main chemical shift assignments for each fraction are detailed in Table 4, and the spectra are provided in the Supplementary Materials (Figures S2–S5). The NMR data of the N series revealed the phase separation patterns of the basic non-polar backbones along the ethanol precipitation gradient. The anomeric proton (H-1) signals of the neutral samples were primarily distributed in the δ 5.10–5.80 region, with corresponding anomeric carbon (C-1) signals concentrated between δ 95.0 and 100.0. This classic chemical shift interval is a core criterion for determining that the macromolecular backbone is dominated by α-configured glycosidic bonds [55]. In the 60 °C extraction system, the ethanol gradient significantly regulated the fractional retention of chain segments. Specifically, the spectrum of U63N (30% ethanol) exhibited characteristic signals of N-methylamino sugars (δ_C_ 38.7, δ_H_ 2.96) [56]. U65N (50% ethanol) showed substituted carbon signals at δ 77.5 and 76.8, while the C-6 hydroxymethyl carbon (δ 60.5) remained in a free state; combined with proton integration, this suggests a core backbone of α-1,4-D-glucan. When the ethanol concentration increased to 80%, the anomeric carbon region of U68N expanded to eight resonance signals (δ 91.9–99.9). Additionally, 6-deoxy sugar methyl signals (δ_C_ 20.0, δ_H_ 1.70–1.39) and deoxymethylene signals (δ 2.42–2.21) emerged in the high-field region, indicating that the extremely low-dielectric-constant environment effectively retained more complex heteropolysaccharide fragments.

The polar P fractions exhibited distinct spectroscopic features compared to the N series. In the 60 °C system, the low- and medium-ethanol fractions (U63P and U65P) displayed relatively regular sugar ring backbones. Their anomeric signals with small coupling constants (around δ 5.22) indicate that the polar molecules at these gradients primarily precipitated depending on the spatial configurations of the long macromolecular chains [57]. However, the 80% ethanol fraction U68P exhibited highly modified characteristics. Strong carbonyl carbon signals at δ 174.9 and 176.4 in the low-field region of the ^13^C spectrum confirmed the presence of uronic acids and acetyl groups, while the δ 20.0–22.0 region verified O-acetylation or N-acetylation modifications [58,59]. Furthermore, a quaternary carbon signal at δ 137.5, a methine alkene carbon signal at δ 111.4, and an amino sugar C-2 specific signal at δ 53.1 were distinctly detected in U68P. This further corroborates the previous conclusion that high-concentration ethanol precipitation targets and enriches specialized polar fragments.

### 3.9. In Vitro Antioxidant Activity

The in vitro antioxidant activities of the 12 polysaccharide fractions from *U. unicinctus* coelomic fluid were evaluated using DPPH, ABTS, and hydroxyl radical (·OH) scavenging models (Table 5). The antioxidant capacity of the polysaccharide fractions was significantly influenced by their acidic nature, extraction temperature, and ethanol precipitation concentration. The acidic (P) fractions consistently outperformed the neutral (N) fractions, particularly in the ·OH assay where only the P-fractions reached measurable IC_50_ values. For instance, U68P exhibited the most potent activity across all models, whereas many neutral fractions failed to show significant inhibition. This enhanced bioactivity is likely attributable to the presence of uronic acid groups in the acidic fractions, which facilitate electron donation and the stabilization of free radicals [60,61]. Furthermore, the higher IC_50_ values observed in fractions extracted at 100 °C compared to 60 °C suggest that elevated temperatures may induce structural changes or thermal degradation of bioactive components, thereby reducing their radical-scavenging efficiency.

Specifically, horizontal comparisons with existing literature regarding DPPH scavenging capability reveal that the hydrogen-donating activity of this polar fraction (U68P, IC_50_ = 2.58 mg/mL) is not only superior to various marine bivalve extracts [62,63] and protein-polysaccharide complexes [64,65], but is also on par with, or even superior to, the highly active purified polysaccharides from the sea cucumber *Apostichopus japonicus* (IC_50_ = 3.11 mg/mL) [66,67]. The ABTS efficacy scavenging of U68P far exceeds that of neutral polysaccharide extracts from various marine bivalves [66,68] and marine polysaccharide-protein conjugates [64,65], and is significantly superior to sulfated glycosaminoglycan hydrolysates derived from abalone viscera [69]. This pronounced superiority in single-electron transfer validates the structural deductions made earlier.

Benchmarking the antioxidant efficacy of the polar fractions in this study against existing marine benthic animal polysaccharides revealed that the ·OH scavenging activities of U18P and U68P were not only significantly superior to various crude oyster polysaccharides and mixed fractions from different extraction stages [66], but also highly comparable to reported neutral purified oyster polysaccharides (IC_50_ = 2.68 mg/mL) [68]. These align with the well-established structure–activity relationships of marine benthic animal polysaccharides, wherein enriched acidic groups (e.g., uronic acid and sulfate groups) on the polysaccharide chains act as the core active centers conferring antioxidant efficacy [60,61]. It is important to note that in vitro chemical assays do not fully replicate the complex physiological environment of cellular or in vivo systems [70]. In the present study, these assays served primarily as preliminary screening tools to complement the neuroprotective findings. Given that neuroprotection is fundamentally linked to redox-regulating mechanisms, the chemical results provide a baseline characterization of the polysaccharides’ radical-scavenging potential before evaluating their efficacy in biological models.

### 3.10. In Vitro Cytotoxicity and Anti-Hypoxic Effects

#### 3.10.1. Cytotoxicity

**Figure 6:**
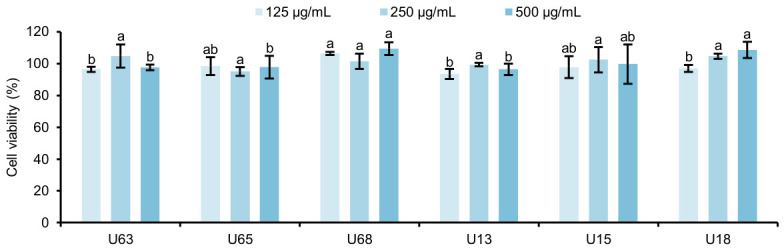
Effects of polysaccharides from the coelomic fluid of *U. unicinctus* on the viability of SH-SY5Y cells. Note: Data are presented as the mean ± SD (*n* = 3). Different lowercase letters above the bars indicate significant differences between groups (*p* < 0.05), while sharing the same letter indicates no significant difference.

Prior to establishing the in vitro experiment to evaluate anti-hypoxia activity, the primary prerequisite is to exclude potential toxic interference from the tested polysaccharides on SH-SY5Y cells, thereby establishing a scientifically sound and safe dosage window. The CCK-8 cell viability assay results (Figure 6) revealed that within the tested concentration gradient of 125–500 μg/mL, the survival rates of SH-SY5Y cells did not decrease significantly after co-incubation with the polysaccharide fractions. The cell viabilities were maintained above 95% of the control group (*p* > 0.05). This result clearly demonstrates that the *U. unicinctus* coelomic fluid polysaccharides possess biocompatibility within this concentration range.

#### 3.10.2. Anti-Hypoxic Effects

**Figure 7:**
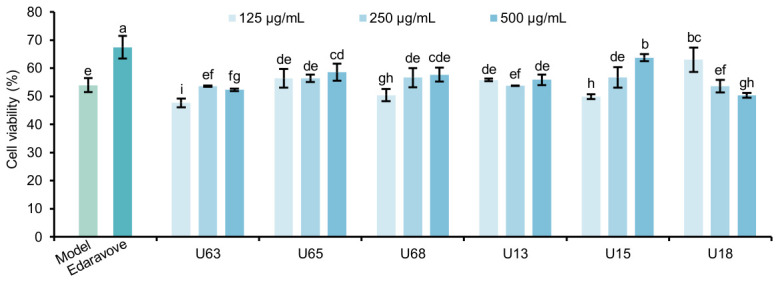
Effects of polysaccharides from the coelomic fluid of *U. unicinctus* on the viability of SH-SY5Y cells under oxygen-glucose deprivation/reoxygenation conditions. Note: Data are presented as the mean ± SD (*n* = 3). Different lowercase letters above the bars indicate significant differences between groups (*p* < 0.05), while sharing the same letter indicates no significant difference. Model: hypoxia model group.

Following the physical OGD/R modeling, the cell viability of the model group dropped significantly to 53.94% (Figure 7). This confirms that severe hypoxia–ischemia induced cellular damage, indicating the establishment of the OGD/R model. Concurrently, treatment with the positive control (edaravone) significantly restored cell viability to 67.42 ± 4.04% (labeled as a, *p* < 0.05), further validating the reliability of the screening assay. In the intervention tests, the six crude polysaccharide fractions prepared via different extraction processes exhibited significant divergence in their neuroprotective efficacy. Notably, the medium- and high-ethanol-precipitated fractions extracted at 100 °C (U15 and U18) demonstrated the most prominent cell-rescue effects. Specifically, U15 at 500 μg/mL significantly restored cell viability to 63.45%. Furthermore, U18 exhibited a potent protective effect at a low dose of only 125 μg/mL, promoting cell viability recovery to 62.95%. In contrast, although the 60 °C extraction group (U65, U68) showed a slight trend of viability recovery (56.35–57.10%) at a high dose of 500 μg/mL, this did not reach statistical significance. The low-ethanol-precipitated macromolecular fraction U63 lacked protective activity entirely. Although the cytoprotective efficacies of these current crude polysaccharide fractions have not yet fully reached the level of the clinical drug edaravone, they demonstrate profound therapeutic potential. Future efforts focusing on the targeted screening of specific active structural motifs or the application of precise chemical modifications are expected to yield polysaccharide derivatives with superior neuroprotective activities.

This significant divergence observed at the macromolecular pharmacodynamics level might corroborate the detailed chemical structural characterizations presented earlier. The excellent anti-hypoxia protective activities demonstrated by U15 and U18 in the OGD/R model indicate that their core material basis lies in the internally enriched specialized polar fragments (e.g., U15P and U18P, as analyzed previously). Having undergone thermodynamic degradation at 100 °C, the weight-average molecular weights of these fractions were significantly reduced, and the high-concentration ethanol precipitation substantially enriched complex heteropolysaccharide structures carrying high-density sulfate groups and amino sugars. Therefore, it is likely that U15 and U18, leveraging the superior antioxidant efficacy of these polar subgroups, effectively block the intracellular ROS burst and oxidative stress cascade induced by OGD/R, ultimately exerting critical neuroprotective effects [71,72].

**Figure 8:**
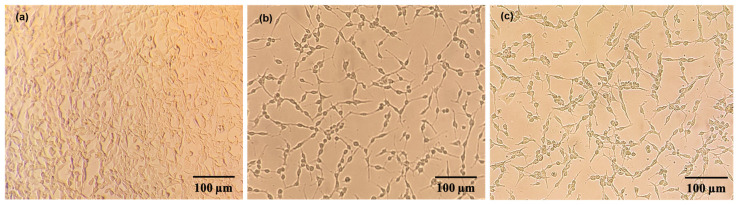
Morphology of the anti-hypoxic effects on neuroblastoma cells. (**a**) Control group; (**b**) model group; and (**c**) treatment with crude polysaccharides from the coelomic fluid of *Urechis unicinctus* obtained by 80% ethanol precipitation (125 µg/mL).

Microscopic morphological observations using an inverted microscope provided direct qualitative supplementary evidence for the quantitative survival rate data obtained via the CCK-8 assay (Figure 8). Cells in the control group exhibited adherence, possessing plump cell bodies with typical spindle or polygonal shapes. Their neurite networks were clearly intertwined, and the overall cell proliferation density was high. Following the physical OGD/R modeling, the microscopic morphology of the cells in the model group underwent significant pathological degeneration. The adherent cell population shrank drastically, accompanied by numerous dead cells floating in the field of view due to the loss of extracellular matrix adhesion. In contrast, the intervention group pretreated with 125 μg/mL U18 crude polysaccharides exhibited a significant reversal and improvement in pathological morphological features compared to the model group. The absolute density of adherent cells in the field of view recovered noticeably, and some cells successfully maintained the physical integrity of their short neurite structures. The degrees of cell body shrinkage and margin passivation were substantially alleviated. Although the overall cell density did not fully recover to the level of the control group, this intuitive representation of morphological improvement highly aligns with the trajectory of quantitative cell survival rates determined by the CCK-8 assay, further confirming the anti-hypoxia neuroprotective efficacy of the *U. unicinctus* coelomic fluid polysaccharides at the cellular phenotypic level.

### 3.11. Structure–Activity Relationship Analysis and Comprehensive Mechanistic Discussion

Molecular weight plays a key role in determining bioactivity. Low-molecular-weight fractions obtained at high ethanol concentrations (e.g., U68P and U18P, ~10^3^ Da) exhibited superior antioxidant and neuroprotective effects compared to high-molecular-weight counterparts (~10^5^ Da). This trend may be attributed to reduced steric hindrance and potentially improved cellular uptake, which could enable more efficient interactions with intracellular targets under hypoxic stress [73]. The degree of sulfation is another factor that correlated strongly with functional performance. Acidic fractions (P fractions), particularly U18P with the highest sulfate content (~24%), showed the most robust activities. It is hypothesized that sulfate groups introduce dense negative charges, which may enhance electrostatic interactions with biomolecules and promote an extended chain conformation. Such a conformation could increase the exposure of active sites, thereby improving radical scavenging and receptor binding [74,75]. Monosaccharide composition further influences functionality. Homoglucan-dominant fractions (e.g., U13N, U15N) exhibited relatively lower activity, whereas highly active fractions (U68P, U18P) contained diverse monosaccharides such as galactose, fucose, and glucosamine. These residues, typical of bioactive marine heteropolysaccharides, may provide additional sites for sulfation and branching, enhancing molecular recognition and bioactivity [76].

In addition, branching degree and backbone architecture likely influence functional performance. Fractions precipitated at high ethanol concentrations appeared enriched in heteropolysaccharide fragments with shorter main chains and higher branching degrees. Such architectures could increase the density of terminal residues and functional groups, which are often the primary sites for biological interactions [77]. The observed increased branching is suggested to contribute to a more flexible and dynamic conformation, potentially allowing for better adaptation to the cellular microenvironment. Finally, microstructural morphology and conformation further modulate these interactions. SEM analysis demonstrated that highly sulfated acidic fractions possess porous, sponge-like three-dimensional structures, whereas neutral fractions exhibit compact and smooth morphologies. The porous architecture is expected to increase the specific surface area, potentially facilitating enhanced interaction with reactive oxygen species and cellular components [73].

Taken together, our data suggest that these structural features may act synergistically to enhance cellular uptake, molecular recognition, and reactive site accessibility. While high-molecular-weight, low-sulfation homoglucans with simple linear structures show comparatively weaker effects, the enhanced activity of the acidic fractions appears to be linked to their complex, highly sulfated, and branched architectures. This analysis provides a preliminary rational framework for the targeted design of anti-hypoxic therapeutic agents, though further studies involving specific structural modifications are necessary to definitively establish these causal relationships.

## 4. Conclusions and Future Perspectives

This study systematically investigated polysaccharides from the coelomic fluid of *U. unicinctus*. Fractionation via gradient ethanol precipitation and ion exchange chromatography yielded distinct populations, wherein high-ethanol fractions were enriched in low-molecular-weight, highly sulfated heteropolysaccharides (comprising galactose, fucose, and glucosamine). These fractions exhibited superior antioxidant and neuroprotective effects in the OGD/R model. Structure–activity relationship analysis confirmed that reduced molecular weight, elevated sulfation, and more diverse monosaccharide composition synergistically enhance bioactivity by improving molecular accessibility and functional group exposure.

While these findings are promising, future research must address remaining structural and mechanistic gaps. Fine structural elucidation using 2D-NMR and mass spectrometry, alongside controlled depolymerization, is necessary to resolve specific motifs like glycosidic linkages and sulfation sites. Furthermore, since physiological (age, life cycle) and environmental (temperature, nutrients) factors influence biosynthesis, subsequent studies should investigate how these variables dictate batch-to-batch structural variations. Finally, comprehensive in vivo validations, pharmacokinetic evaluations, and targeted studies on oxidative stress, mitochondrial function, and apoptosis regulation are essential to confirm their specific anti-hypoxic mechanisms and fully assess their therapeutic potential.

## Figures and Tables

**Table 1:** Parameters of polysaccharides extracted from the coelomic fluid of *U. unicinctus* under different conditions.

Sample	Temperature (°C)	EtOH (%)	Yield (‰)	Protein (%)	Carbohydrate (%)	N Fractions (%)	P Fractions (%)
U63	60	30	3.80 ± 0.05 a	2.35 ± 0.04 a	94.75 ± 0.30 ab	10.78 ± 1.82 a	78.42 ± 1.95 a
U65	60	50	3.52 ± 0.04 b	2.03 ± 0.08 b	91.72 ± 1.15 cd	23.56 ± 2.41 b	66.94 ± 3.05 b
U68	60	80	2.85 ± 0.02 c	1.60 ± 0.06 c	94.18 ± 0.52 abc	38.06 ± 3.12 c	54.04 ± 2.88 c
U13	100	30	2.11 ± 0.09 d	0.87 ± 0.03 e	90.94 ± 1.39 d	9.22 ± 1.45 a	79.28 ± 2.13 a
U15	100	50	1.85 ± 0.02 e	0.94 ± 0.05 e	95.68 ± 1.13 a	48.61 ± 2.76 d	46.59 ± 3.42 d
U18	100	80	1.51 ± 0.02 f	1.23 ± 0.03 d	90.99 ± 1.03 d	46.72 ± 3.55 d	38.88 ± 2.91 e

Note: Data in the table are expressed as mean ± standard deviation; different lowercase letters within the same column indicate significant differences (*p* < 0.05); all parameters are calculated based on the wet weight of coelomic fluid.

**Table 2:** Monosaccharide composition of polysaccharides from the coelomic fluid of *U. unicinctus*.

Sample	Fuc (%)	Ara (%)	GlcN (%)	Gal (%)	Glc (%)	Man (%)	Rib (%)	Other (%)
U63N	1.01	–	–	–	93.91	–	2.11	2.97
U65N	0.44	–	–	–	99.56	–	–	–
U68N	2.25	–	5.37	2.82	76.86	4.48	8.22	–
U13N	0.43	–	–	–	99.57	–	–	–
U15N	0.28	–	–	–	99.72	–	–	–
U18N	2.32	–	4.66	5	72.92	3.95	11.15	–
U63P	0.44	–	–	–	95.93	–	2.24	1.39
U65P	–	–	–	–	96.56	–	1.44	2
U68P	14.14	2.74	10.68	32.25	6.73	9.77	21.42	2.27
U13P	1.18	-	–	0.99	89.98	1.15	4.25	2.45
U15P	–	-	–	–	94.78	–	2.29	2.93
U18P	13.29	-	10.31	32.76	9.25	10.32	24.07	–

Note: Fuc, fucose; Ara, arabinose; GlcN, glucosamine; Gal, galactose; Glc, glucose; Man, mannose; Rib, ribose. Among the samples, U63, U65, and U68 represent those extracted at 60 °C and obtained by alcohol precipitation at concentrations of 30%, 50%, and 80%, respectively; U13, U15, and U18 represent those extracted at 100 °C and obtained by alcohol precipitation at concentrations of 30%, 50%, and 80%, respectively. N and P denote the neutral and acidic (polar) polysaccharide fractions, respectively. “–” denotes data was not obtained.

**Table 3:** Molecular weight distribution of polysaccharides from the coelomic fluid of *U. unicinctus*.

Sample	*M*_p_ (Da)	*M*_n_ (Da)	*M*_w_ (Da)	*M*_z_ (Da)	*M*_z+1_ (Da)	PDI
U63N	1.81 × 10^5^ a	8.14 × 10^4^ a	1.29 × 10^5^ a	1.72 × 10^5^ a	2.03 × 10^5^ a	1.591
U65N	1.63 × 10^5^ a	6.98 × 10^4^ a	1.12 × 10^5^ a	1.66 × 10^5^ a	2.34 × 10^5^ a	1.606
U68N	1.68 × 10^3^ b	1.60 × 10^3^ b	2.00 × 10^3^ b	2.45 × 10^3^ b	2.88 × 10^3^ b	1.248
U13N	1.47 × 10^5^ a	6.13 × 10^4^ a	9.53 × 10^4^ a	1.30 × 10^5^ a	1.57 × 10^5^ a	1.554
U15N	1.46 × 10^5^ a	6.97 × 10^4^ a	1.04 × 10^5^ a	1.39 × 10^5^ a	1.71 × 10^5^ a	1.494
U18N	1.55 × 10^3^ b	1.55 × 10^3^ b	1.93 × 10^3^ b	2.37 × 10^3^ b	2.80 × 10^3^ b	1.240
U63P	1.68 × 10^5^ a	7.36 × 10^4^ a	1.22 × 10^5^ a	1.71 × 10^5^ a	2.06 × 10^5^ a	1.663
U65P	1.78 × 10^5^ a	7.35 × 10^4^ a	1.23 × 10^5^ a	1.73 × 10^5^ a	2.10 × 10^5^ a	1.673
U68P	2.12 × 10^3^ b	1.72 × 10^3^ b	2.17 × 10^3^ b	2.64 × 10^3^ b	3.05 × 10^3^ b	1.257
U13P	1.63 × 10^5^ a	6.45 × 10^4^ a	1.07 × 10^5^ a	1.57 × 10^5^ a	2.06 × 10^5^ a	1.661
U15P	1.70 × 10^5^ a	7.23 × 10^4^ a	1.20 × 10^5^ a	1.73 × 10^5^ a	2.20 × 10^5^ a	1.660
U18P	2.00 × 10^3^ b	1.69 × 10^3^ b	2.13 × 10^3^ b	2.60 × 10^3^ b	3.01 × 10^3^ b	1.256

Note: *M*_p_, peak molecular weight; *M*_n_, number-average molecular weight; *M*_w_, weight-average molecular weight; *M*_z**,**_ z-average molecular weight; *M*_z+1_, z + 1-average molecular weight; PDI, polydispersity index (*M*_w_/*M*_n_). Values within the same column followed by different letters indicate significant differences (*p* < 0.05). U63, U65, and U68 represent those extracted at 60 °C and obtained by alcohol precipitation at concentrations of 30%, 50%, and 80%, respectively; U13, U15, and U18 represent those extracted at 100 °C and obtained by alcohol precipitation at concentrations of 30%, 50%, and 80%, respectively. N and P denote the neutral and acidic (polar) polysaccharide fractions, respectively.

**Table 4:** Major ^1^H and ^13^C NMR chemical shift assignments of polysaccharides from the coelomic fluid of *U*. *unicinctus*.

Sample	Anomeric Region (C-1/H-1)	Sugar Ring Skeleton (C-2~C-5/H-2~H-5)	Hydroxymethyl Group (C-6/H-6)	Side Chains and Characteristic Groups
U63P	^13^C: 99.9 ^1^H: 5.70–5.51, 5.22	^13^C: 77.6, 76.8 (sub.), 73.3–69.3 ^1^H: 4.38–3.69	^13^C: 60.5 ^1^H: 4.38–3.82 (m)	N.D.
U65P	^13^C: 99.9, 98.5 ^1^H: 5.75–5.48, 5.23	^13^C: 77.2, 73.3–69.3 ^1^H: 4.36–3.69	^13^C: 60.5 ^1^H: 4.36–3.77 (m)	N.D.
U68P	^13^C: 95.2, 86.4, 85.0, 82.3 ^1^H: 5.82–5.21 (incl. alkene ^1^H), 4.58–4.30	^13^C: 72.5–65.9 ^1^H: 4.27–3.33	^13^C: 62.4, 61.2, 61.1, 60.1, 59.9 ^1^H: 4.27–3.81 (m)	Carbonyl (C=O): ^13^C 176.4, 174.9 Alkene (C=C): ^13^C 137.5, 111.4 Amino C_2_: ^13^C 53.1 Ac–CH_3_: ^13^C 22.0–20.0; ^1^H 2.55–2.05 Deoxy CH_3_: ^13^C 15.0, 11.5; ^1^H 1.70–0.94
U13P	^13^C: 99.8, 98.5 ^1^H: 5.60, 5.27–5.15	^13^C: 77.1, 73.3–70.3 ^1^H: 4.38–3.65	^13^C: 69.3 (sub.), 60.5 (unsub.) ^1^H: 4.38–3.65 (m)	N.D.
U15P	^13^C: 100.4, 100.2, 99.6, 97.8 ^1^H: 5.53–5.17	^13^C: 82.3 (sub.), 72.6–66.9 ^1^H: 4.55–3.46	^13^C: 61.1, 59.9 ^1^H: 4.55–3.46 (m)	Carbonyl (C=O): ^13^C 176.4 Ac–CH_3_: ^13^C 22.2, 22.0; ^1^H 2.52–2.11 Deoxy CH_3_: ^13^C 15.0; ^1^H 1.72–1.08
U18P	^13^C: 99.8, 98.5, 97.9 ^1^H: 5.61, 5.23	^13^C: 77.6, 76.9, 73.3–69.3 ^1^H: 4.41–3.69	^13^C: 60.5 ^1^H: 4.41–3.82 (m)	N.D.
U63N	^13^C: 99.9, 99.7, 99.6, 98.6, 97.9, 95.7 ^1^H: 5.73–5.50, 5.26–5.11	^13^C: 77.5–68.5 ^1^H: 4.34–3.67	^13^C: 62.4, 60.5 ^1^H: 4.34–3.80 (m)	N–CH_3_: ^13^C 38.7; ^1^H 2.96
U65N	^13^C: 99.9, 99.8, 98.6 ^1^H: 5.80–5.50, 5.31–5.13	^13^C: 77.5, 76.8 (sub.), 74.0–69.3 ^1^H: 4.32–3.68	^13^C: 60.5 ^1^H: 4.32–3.79 (m)	N.D.
U68N	^13^C: 99.9–91.9 (8 signals) ^1^H: 5.67–5.50, 5.26–5.15	^13^C: 77.0–68.5 ^1^H: 4.12–3.68	^13^C: 61.0, 60.6, 60.5, 60.4 ^1^H: 4.12–3.68 (m)	Deoxy CH_3_: ^13^C 20.0; ^1^H 1.70–1.39 Deoxy CH_2_: ^1^H 2.42–2.21
U13N	^13^C: 100.0, 99.8 ^1^H: 5.73–5.52, 5.29–5.13	^13^C: 76.8 (sub.), 73.3–68.5 ^1^H: 4.35–3.64	^13^C: 60.4 ^1^H: 3.91	Deoxy CH_3_: ^13^C 20.0
U15N	^13^C: 99.9, 99.7, 99.6, 98.5, 98.0 ^1^H: 5.60, 5.27–5.16	^13^C: 77.4–69.3 ^1^H: 4.41–3.68	^13^C: 60.5, 60.4 ^1^H: 4.41–3.80 (m)	Deoxy CH_3_: ^13^C 20.0 Deoxy CH_2_: ^1^H 2.30, 1.61–1.36
U18N	^13^C: 99.9, 99.7, 99.6 ^1^H: 5.61, 5.21	^13^C: 74.0–69.3 ^1^H: 4.42–3.61	^13^C: 60.6, 60.5, 60.4 ^1^H: 4.42–3.77 (m)	–CH_2_–: ^13^C 20.0; ^1^H 2.42–2.21, 1.66–1.25

Note: All NMR spectra were recorded in D_2_O as the solvent. The ^1^H NMR frequency was 600 MHz, and the ^13^C NMR frequency was 151 MHz. N.D., not detected; (m), multiplet, indicating overlapping of proton signals with other methine protons of the sugar ring; (sub.), substituted carbon signal at this position; Ac-CH_3_, acetyl methyl group; C=O, carboxyl carbon of uronic acid or carbonyl carbon of acetyl ester; N-CH_3_, N-methyl group.

**Table 5:** IC_50_ values of polysaccharide fractions in three antioxidant models.

Sample	DPPH (mg/mL)	ABTS (mg/mL)	OH (mg/mL)
U63N	3.54 ± 0.35 f	5.35 ± 0.92 b	N/A
U65N	4.18 ± 0.51 ef	8.42 ± 2.14 a	N/A
U68N	5.15 ± 0.88 d	N/A	N/A
U13N	5.41 ± 0.19 d	6.21 ± 1.05 b	N/A
U15N	8.06 ± 0.64 a	N/A	N/A
U18N	4.92 ± 0.28 de	10.15 ± 3.20 a	N/A
U63P	6.48 ± 0.42 c	4.26 ± 0.41 cd	N/A
U65P	7.45 ± 0.38 b	4.48 ± 0.76 c	N/A
U68P	2.58 ± 0.12 g	0.48 ± 0.05 g	2.31 ± 0.18 d
U13P	6.92 ± 0.23 bc	2.44 ± 0.28 e	4.15 ± 0.85 a
U15P	7.68 ± 0.21 ab	3.51 ± 0.32 d	3.58 ± 0.41 b
U18P	5.11 ± 0.44 d	1.52 ± 0.14 f	2.84 ± 0.22 c

Note: Values within the same column followed by different lowercase letters indicate significant differences (*p* < 0.05). N/A, not applicable, which indicates that the IC_50_ value was not acquired.

## Data Availability

The original contributions presented in this study are included in the article/Supplementary Material. Further inquiries can be directed to the corresponding authors.

## Supplementary Materials

The following supporting information can be downloaded at https://www.mdpi.com/article/10.3390/polym18101203/s1: Figure S1: HPAEC-PAD chromatograms of ten monosaccharide and amino sugar standards; Table S1: Assignments of characteristic FT-IR absorption bands for polysaccharides from the coelomic fluid of *U. unicinctus*; Figure S2: NMR spectra of the polar polysaccharides extracted with 60 °C temperature; Figure S3: NMR spectra of the polar polysaccharides extracted with 100 °C temperature; Figure S4. NMR spectra of the neutral polysaccharides extracted at 60 °C; Figure S5. NMR spectra of the neutral polysaccharides extracted at 100 °C.
