# Supplementary material for: Polysaccharides from the Coelomic Fluid of Urechis unicinctus: Extraction, Structural Diversity, and Potential Against Hypoxia
Source: Polymers (Basel). 2026 May 14;18(10):1203. doi: 10.3390/polym18101203 (PMC13210939; doi:10.3390/polym18101203)
Supplement: Supplementary file 1 [file polymers-18-01203-s001.zip › polymers-4302224-supplementary.pdf]

Supplementary Materials

Polysaccharides from the Coelomic Fluid of *Urechis unicinctus*: Extraction, Structural Diversity, and Potential Against Hypoxia

Xiaodi Wang [1,2], Wenjie Wang [1,3], Rongfeng Li [1,3], Kun Gao [1,3], Ronge Xing [1,3], Xuexin Zhang [1,2], Gaoli Zhou [1,2], Lijing Yin [1,2], Junhao Chen [1,2], Hang Li [1,4,*] and Guantian Li [1,4,*]

[1] Laboratory of Experimental Marine Biology, Institute of Oceanology, Chinese Academy of Sciences, Qingdao 266000, China

[2] University of Chinese Academy of Sciences, Beijing 100049, China

[3] Laboratory for Marine Drugs and Bioproducts, Qingdao Marine Science and Technology Center, Qingdao 266237, China

[4] Laboratory for Marine Biology and Biotechnology, Qingdao Marine Science and Technology Center, Qingdao 266237, China

* Correspondence: hangli@qdio.ac.cn (H.L.); guantianli@qdio.ac.cn (G.L.)

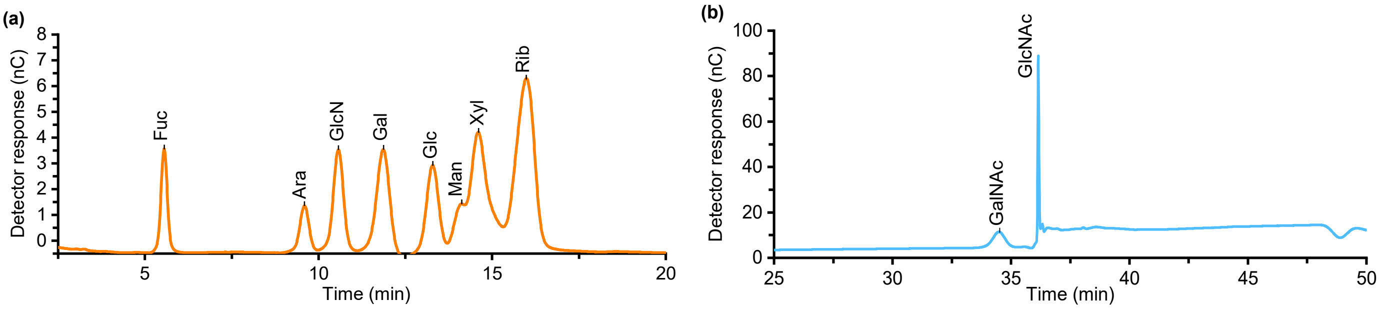

Figure S1. HPAEC-PAD chromatograms of ten monosaccharide and amino sugar standards. (a) The separation of neutral monosaccharides and deoxy-monosaccharides (0–20 min); (b) The separation of acetylated amino sugars (25–50 min).

Abbreviations: Fuc, fucose; Ara, arabose; GlcN, glucosamine; Gal, galactose; Glc, glucose; Man, mannose; Xyl, xylose; Rib, ribose; GalNAc, N-acetylgalactosamine; GlcNAc, N-acetylglucosamine.

Table S1. Assignments of characteristic FT-IR absorption bands for polysaccharides from the coelomic fluid of *U. unicinctus*.

Sample	O−H	C−H	Amide I / Water	C−H bending	S=O stretching	C−O−C skeleton	α-glycosidic
U63N	3294	2924	1643	1413	1242	1024	848
U65N	3307	2925	1642	1415	1245	1024	846
U68N	3326	2931	1648	1419	1240	1026	842
U13N	3315	2928	1645	1416	1243	1025	845
U15N	3310	2926	1644	1414	1244	1024	847
U18N	3320	2929	1646	1417	1241	1027	844
U63P	3385	2927	1651	1420	1238	1023	851
U65P	3379	2928	1649	1418	1240	1022	849
U68P	3388	2932	1654	1422	1236	1028	846
U13P	3382	2928	1650	1419	1239	1024	850
U15P	3376	2927	1648	1417	1241	1023	852
U18P	3390	2930	1655	1421	1237	1029	848

Note: All numerical values in the table are expressed in cm^{-1}. O–H: hydroxyl stretching vibration; C–H: aliphatic C–H stretching vibration; S=O: asymmetric stretching vibration of sulfate groups; C–O–C: skeletal vibration of pyranose ring; α-glycosidic: characteristic absorption of α-type glycosidic linkages.

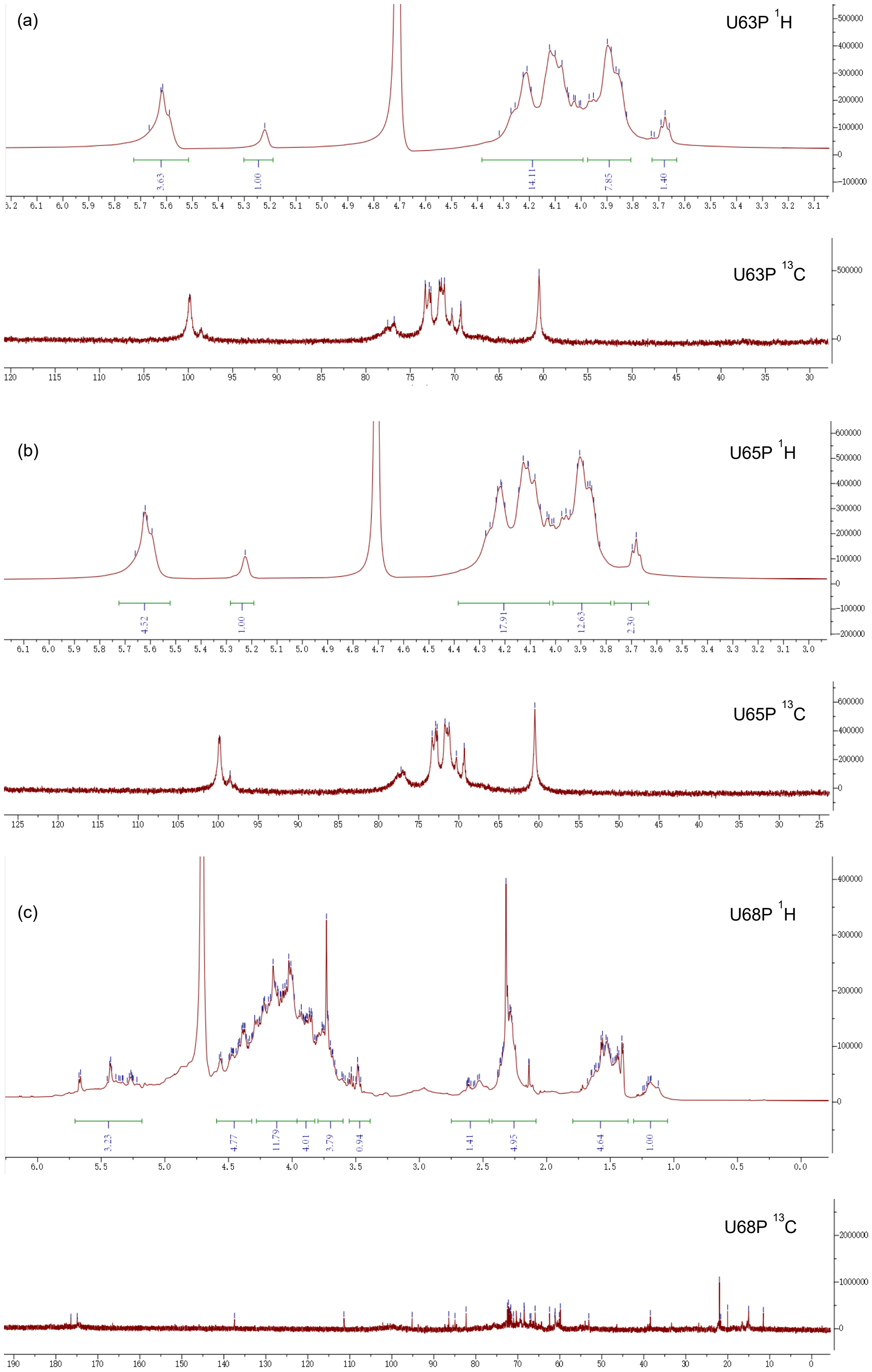

Figure S2. NMR spectra of the polar polysaccharides extracted with 60 °C temperature.

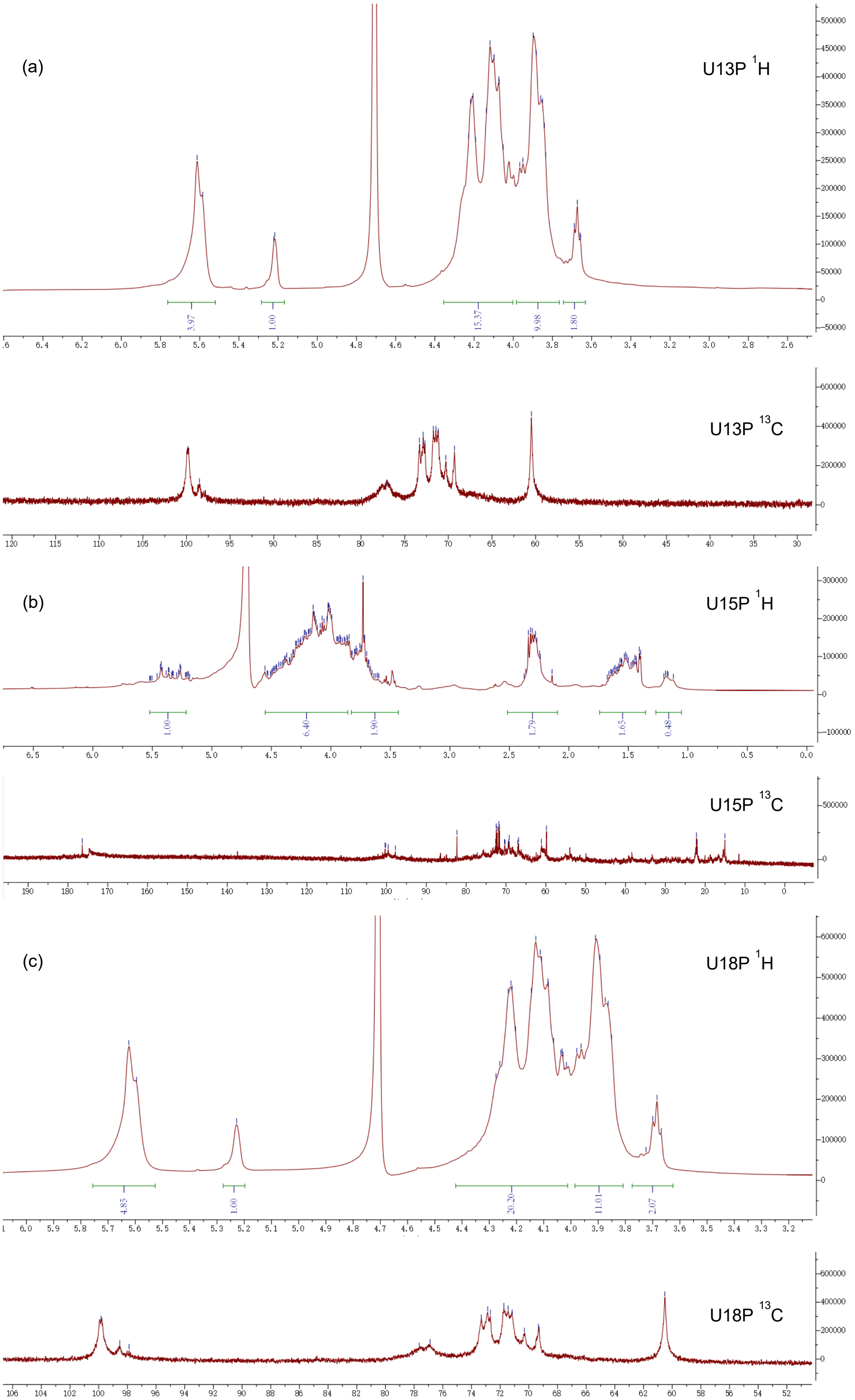

Figure S3. NMR spectra of the polar polysaccharides extracted with 100 °C temperature.

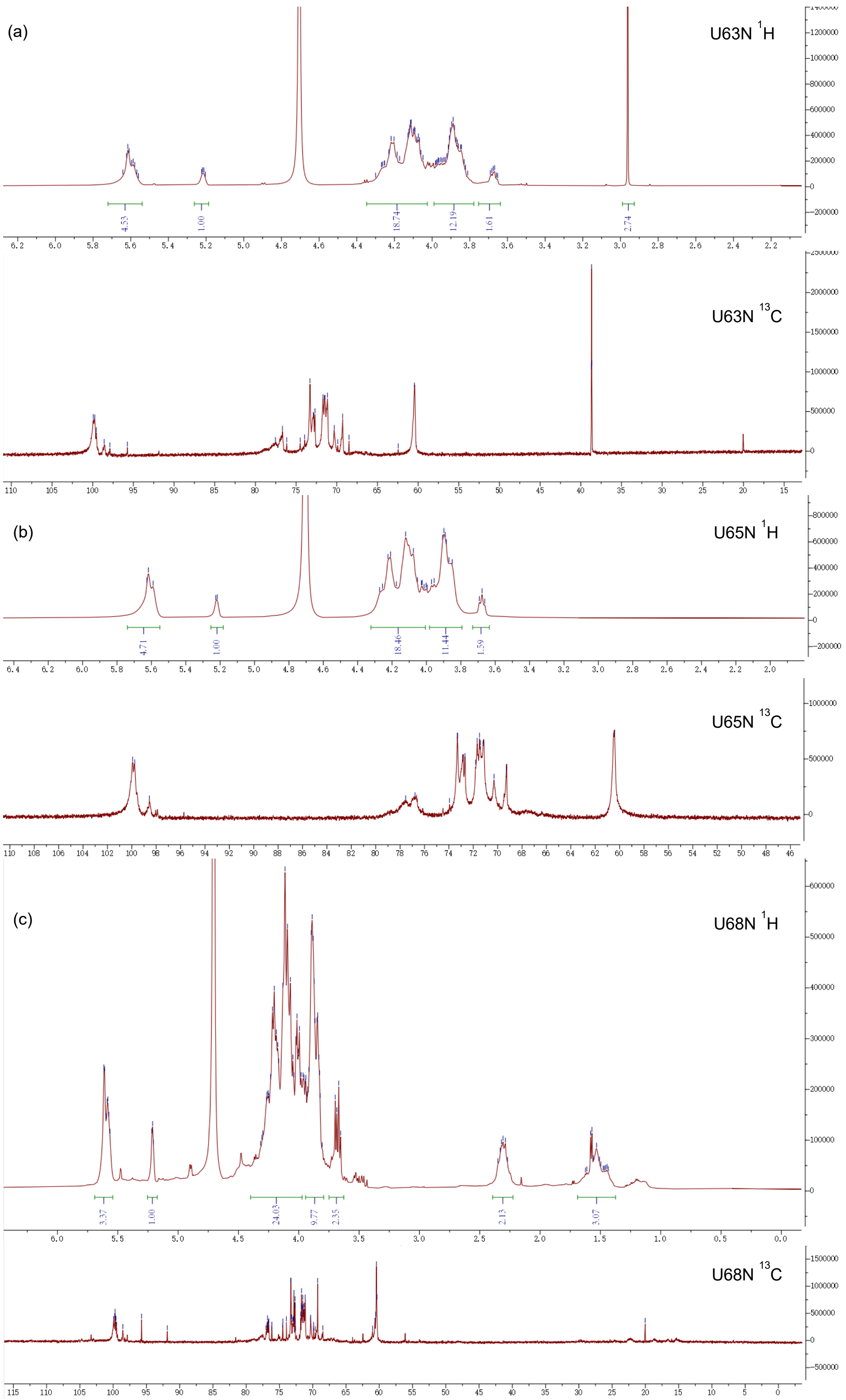

Figure S4. NMR spectra of the neutral polysaccharides extracted with 60 °C temperature.

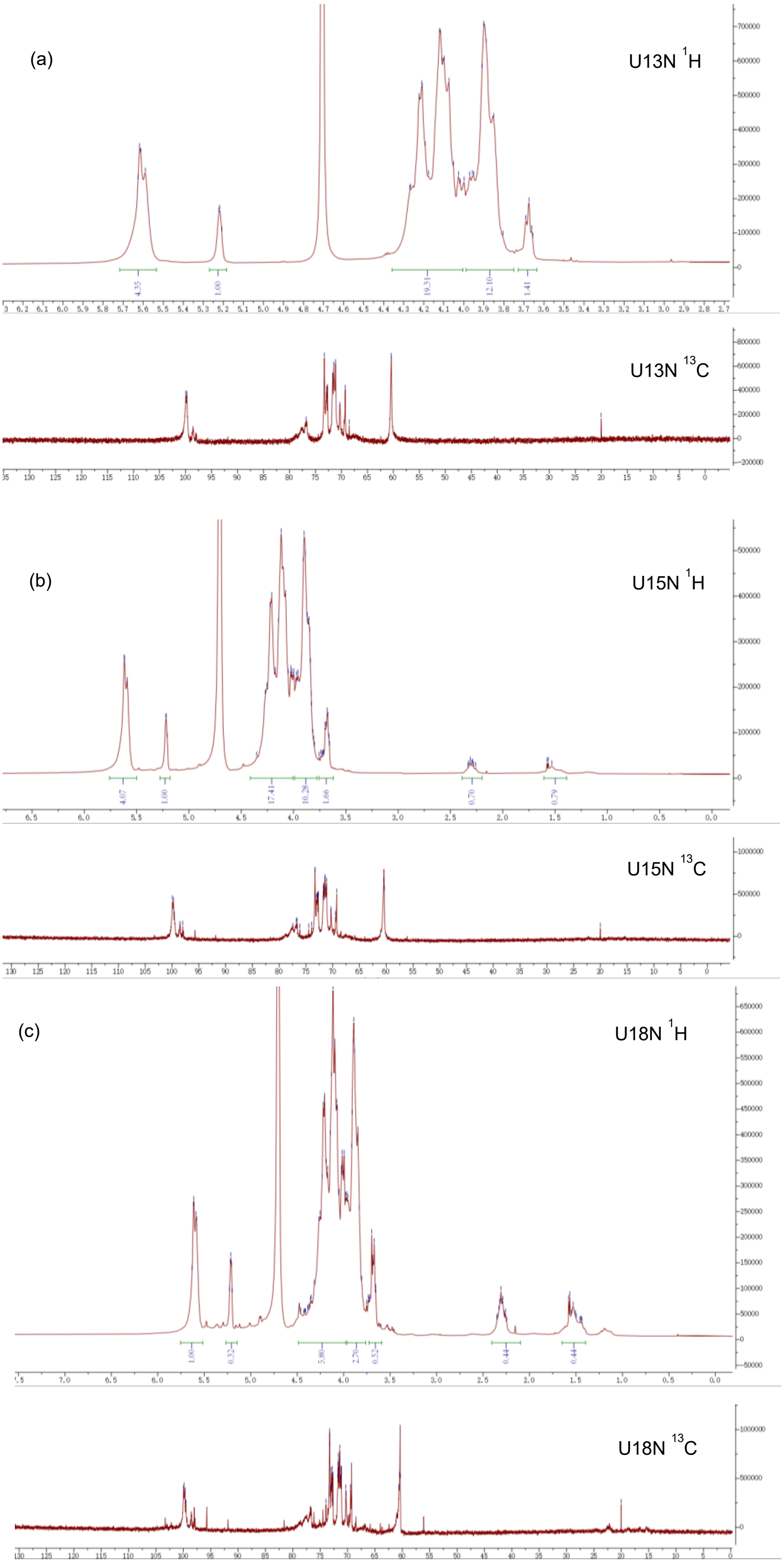

Figure S5. NMR spectra of the neutral polysaccharides extracted with 100 °C temperature.
